# Supplementary material for: RNF138 Downregulates Antiviral Innate Immunity by Inhibiting IRF3 Activation
Source: Int J Mol Sci. 2023 Nov 9;24(22):16110. doi: 10.3390/ijms242216110 (PMC10671598; doi:10.3390/ijms242216110)
Supplement: Supplementary file 1 [file ijms-24-16110-s001.zip › ijms-2662551-supplementary.pdf]

Supplementary information Table S1 Primers for qPCR

Gene	Species	Forward primer (5’-3’)	Reverse primer (5’-3’)
GAPDH	Human	ATGACATCAAGAAGGTGGTG	CATACCAGGAAATGAGCTTG
CXCL10	Human	GCTCTACTGAGGTGCTATGTTC	GGAGGATGGCAGTGGAAGTC
RNF138	Human	CAGACAGCGTTTACTGGATCAC	TGGTAATCTGGCTAGGATCTCC
IFNB1	Human	AGGACAGGATGAACTTTGAC	TGATAGACATTAGCCAGGAG
ISG15	Human	GAGAGGCAGCGAACTCATCTT	CCAGCATCTTCACCGTCAGG
IL-6	Human	ACTCACCTCTTCAGAACGAATTG	CCATCTTTGGAAGGTTCAGGTTG
PTEN	Human	TGGATTCGACTTAGACTTGACCT	GGTGGGTTATGGTCTTCAAAAGG

Supplementary Table S2 Primers for shRNA

shRNF138#1 Forward
CCGGTTTGAACGATGTGATTGATATCTCGAGATATCAATCACATCGTTCAAATTTTTG
shRNF138#1 Reverse
AATTCAAAAATTTGAACGATGTGATTGATATCTCGAGATATCAATCACATCGTTCAAA
shRNF138#2 Forward
CCGGGCTAGATGAAGAAACCCAATACTCGAGTATTGGGTTTCTTCATCTAGCTTTTTG
shRNF138#2 Reverse
AATTCAAAAAGCTAGATGAAGAAACCCAATACTCGAGTATTGGGTTTCTTCATCTAGC
shRNF138#3 Forward
CCGGCCTAGCCAGATTACCAGAAATCTCGAGATTTCTGGTAATCTGGCTAGGTTTTTG
shRNF138#3 Reverse
AATTCAAAAACCTAGCCAGATTACCAGAAATCTCGAGATTTCTGGTAATCTGGCTAGG

Supplementary Table S3 Primers for sgRNA

Gene	Species	Forward primer (5’-3’)	Reverse primer (5’-3’)
RNF138-sgRNA#1	Human	CACCGCAAAACGCCCGTGCGGACCA	AAACTGGTCCGCACGGGCGTTTTG

RNF138-sgRNA#2	Human	CACCGTAGAAATCATCTTCGGTGT	AAACACACCGAAGATGATTTCTAC
Rnf138-sgRNA	Mouse	CACCGTCGGCGGCCACGTCCTACA	AAACTGTAGGACGTGGCCGCCGAC
